# Observation of dynamic nuclear polarization echoes

**DOI:** 10.1126/sciadv.adr2420

**Published:** 2024-10-18

**Authors:** Nino Wili, Anders B. Nielsen, José P. Carvalho, Niels Chr. Nielsen

**Affiliations:** Interdisciplinary Nanoscience Center (iNANO) and Department of Chemistry, Aarhus University, Gustav Wieds Vej 14, DK-8000 Aarhus C, Denmark.

## Abstract

It is demonstrated that the time evolution of the electron-nuclear polarization transfer process during pulsed dynamic nuclear polarization (DNP) can be reversed on a microsecond timescale, leading to the observation of DNP echoes. The DNP echoes are induced by consecutive application of two pulse trains that produce effective Hamiltonians that differ only in the sign of the effective hyperfine coupling. The experiments have been performed on a frozen solution of trityl radicals in water/glycerol on a homebuilt X-band electron paramagnetic resonance/DNP spectrometer at 80 kelvins. We envisage that DNP echoes will play an important role in future development of pulsed DNP for sensitivity-enhanced nuclear magnetic resonance, hyperfine spectroscopy, and quantum sensing.

## Teaser

Time reversal of electron-nuclear spin polarization transfer was achieved by a tailored microwave pulse sequence.

## INTRODUCTION

Echo phenomena are a cornerstone of coherent spectroscopy. They are central to magnetic resonance, including nuclear magnetic resonance (NMR) (*1*, *2*) and electron paramagnetic resonance (EPR) (*3*, *4*), but echoes have also been observed in Ruby lasers (*5*) and in microwave (mw) (*6*) and infrared spectroscopy (*7*). In magnetic resonance, echoes are formed by refocusing an interaction in the spin Hamiltonian that leads to an apparent signal decay. For example, the pioneering Hahn echo (*8*) refocuses heteronuclear spin-spin couplings and inhomogeneities in the external field. Another important example is the magic echo and variants thereof, which have been shown to invert the evolution in a homonuclear system of strongly coupled spins (*9*, *10*). This observation violates the spin temperature hypothesis and highlights that many “relaxation” phenomena in magnetic resonance are coherent in nature and, accordingly, may be dealt with through pulsed operation and refocused at least in principle. This phenomenon has been referred to as “time-reversal” experiments (*11*), as the system evolves as if time was reversed.

Besides spin echoes, also polarization and coherence transfer elements are fundamental both in NMR and EPR (*2*, *4*). In NMR of solids, the most prominent polarization transfer scheme is cross-polarization (CP) (*12*), where two dipolar-coupled heteronuclei are spin locked by radio-frequency fields with the same nutation frequency, known as Hartmann-Hahn matching (*13*). In the context of echoes, it has been shown that it is possible to invert the sign of the effective heteronuclear coupling Hamiltonian by strategically choosing the phases and offsets of both spin locks, leading to the so-called CP echoes (*14*).

In spin systems containing electron spins and nuclear spins, the electron-nuclear polarization transfer is known as dynamic nuclear polarization (DNP) (*15*). Historically, most DNP experiments have been conducted with continuous-wave mw irradiation. This is especially the case for high-field, high-resolution DNP-enhanced NMR aimed at investigating chemical and biological systems with orders of magnitude higher sensitivity than reachable by conventional NMR (*16*, *17*). Pulsed DNP was introduced in 1987 with the nuclear spin orientation via electron spin locking (NOVEL) pulse sequence (*18*, *19*). This experiment is the analogon of the CP experiment, but, because of the large difference between gyromagnetic ratios of electron and nuclear spins, polarization transfer through the pseudosecular hyperfine coupling is, in this case, driven by matching the spin lock Rabi frequency of the electrons to the Larmor frequency of the nuclei. In this particular case, no irradiation of the nuclei is required. In recent years, many other pulsed-DNP schemes have been developed (*20*–*24*), and it was recognized that the description of pulsed-DNP sequences in many respects resembles that of magic angle spinning recoupling sequences (*21*). In the latter case, the pulse sequences interfere with the physical sample rotation. In pulsed DNP, the pulse sequence interferes with the rotation in spin space due to the nuclear Zeeman interaction.

In this work, we show that it is possible to invert the effective hyperfine couplings in pulsed DNP, which leads to the formation of DNP echoes. Such echoes may have important applications in further development of pulsed DNP for atomic structure analysis and emerging quantum technologies. The overall idea is the following: We consider a system of one electron spin and *N_I_* nuclear spins of the same isotope. The Hamiltonian of this system, in the electron-spin rotating frame and using the high-field approximation for the electron spin, is given in angular frequencies by

$$\begin{array}{c} ℋ=\Delta \omega_{S}S_{z}+{ℋ}_{\text{mw}}\\ +\sum_{i}^{N^{I}}\;\omega_{I}I_{iz}+S_{z}\left(A_{x}^{i}I_{\mathrm{ix}}+A_{y}^{i}I_{\mathrm{iy}}+A_{z}^{i}I_{\mathrm{iz}}\right)\\ +{ℋ}_{\text{nn}} \end{array}$$ (1)

where Δω*_S_* is the electron spin offset, ${ℋ}_{\text{mw}}$ describes the mw irradiation, and ω*_I_* = −γ*_I_B*_0_ is the nuclear Zeeman frequency, with γ*_I_* being the gyromagnetic ratio of the nuclei and *B*_0_ being the external magnetic field. The quantity *A^i^* is the anisotropic hyperfine interaction between the electron and nucleus *i*, and ${ℋ}_{\text{nn}}$ corresponds to the nuclear-nuclear couplings.

Our goal is to generate an effective Hamiltonian that leads to electron-nuclear spin polarization transfer and then to invert the sign of the effective Hamiltonian to create echoes that can be observed on either of the two spin species. This should be achieved by irradiating the electrons only. We will restrict ourselves to periodic irradiation schemes, where ${ℋ}_{\text{mw}}(t+\tau_{m})={ℋ}_{\text{mw}}(t)$ with τ_m_ being the period of the mw Hamiltonian. The associated frequency is called the modulation frequency of the sequence, given by ω_m_ = 2π/τ_m_. It was shown by single-spin vector analysis and average Hamiltonian theory (*23*, *25*–*27*) and operator-based Floquet theory (*21*) that the first-order effective Hamiltonian governing the polarization transfer in DNP can be written as

$$\begin{array}{c} {ℋ}_{\text{eff}}=-\omega_{\text{eff}}^{\left(S\right)}{\tilde{S}}_{z}+\omega_{\text{eff}}^{\left(I\right)}I_{z}\\ +\sum_{i}^{N_{I}}\;\frac{B_{i}}{4}\left[\left(a_{\Delta }{\tilde{S}}^{+}I^{-}+a_{\Delta }^{*}{\tilde{S}}^{-}I^{+}\right)\right.\\ +\left.\left(a_{\Sigma }{\tilde{S}}^{+}I^{+}+a_{\Sigma }^{*}{\tilde{S}}^{-}I^{-}\right)\right]\\ +{\tilde{ℋ}}_{\text{nn}} \end{array}$$ (2)

with $B=\sqrt{A_{x}+A_{y}}$. A stand-alone derivation is given in the Supplementary Materials. The tilde indicates an effective frame, where the *z* axis points along the overall axis of rotation over one period of the periodic pulse sequence. The magnitude of the effective fields is given by ω_eff_ = β_eff_/τ_m_, where β_eff_ is the overall angle of rotation. We choose the convention ∣β_eff_ ∣ ≤ π, such that the effective field is at most half the modulation frequency, ∣ω_eff_ ∣ ≤ ω_m_/2 = π/τ_m_. If the nuclear spins are not irradiated, then the nuclear effective field is $\omega_{\text{eff}}^{\left(I\right)}=\omega_{I}-\text{round}\left(\omega_{I}/\omega_{m}\right)\omega_{m}$. The strength of the effective hyperfine coupling is encoded in the scaling factors *a*_Δ_ and *a*_Σ_, which depend on the details of the electron spin trajectory and are complex in general (*23*).

Depending on the size and signs of the electron or nuclear spin effective fields, the polarization can be transferred in the zero-quantum (ZQ) or double-quantum (DQ) subspaces. With the explicit signs given in Eq. 2, ZQ transfer takes place if $\omega_{\text{eff}}^{\left(S\right)}\approx -\omega_{\text{eff}}^{\left(I\right)}$, and DQ transfer takes place if $\omega_{\text{eff}}^{\left(S\right)}\approx \omega_{\text{eff}}^{\left(I\right)}$. Usually, it is desirable that only one of the two takes place, which is the case either if $|\omega_{\text{eff}}^{\left(S\right)}|\approx |\omega_{\text{eff}}^{\left(I\right)}|\gg |B_{i}|$ or if $|\omega_{\text{eff}}^{\left(S\right)}|\approx |\omega_{\text{eff}}^{\left(I\right)}|\approx 0$ and either *a*_Δ_ = 0 or *a*_Σ_ = 0.

Let us assume that we choose a ZQ-resonance condition with a sizable effective field and a negative Zeeman frequency of the nuclei. In this case, the DQ terms in the effective Hamiltonian are truncated, and we are left with

$$\begin{array}{c} {ℋ}_{\text{eff}}=|\omega_{\text{eff}}^{\left(S\right)}|{\tilde{S}}_{z}+|\omega_{\text{eff}}^{\left(I\right)}|I_{z}\\ +\sum_{i}^{N_{I}}\;\frac{B_{i}}{4}\left(a_{\Delta }{\tilde{S}}^{+}I^{-}+a_{\Delta }^{*}{\tilde{S}}^{-}I^{+}\right)\\ +{\tilde{ℋ}}_{\text{nn}} \end{array}$$ (3)

So far, the action of this type of Hamiltonians has essentially only been considered for the ${\tilde{S}}_{z}\to I_{z}$ transfer corresponding to an inversion (π rotation) in the ZQ space. Accordingly, if only these two operators are of interest, then the phase of *a*_Δ_ does not matter. However, for our goal, this phase is essential, because it allows for an inversion of the coupling Hamiltonian. Note that the phase of *a*_Δ_ is defined with respect to the *z* axis of the effective electron spin frame. If the effective field of the electron spins points along the laboratory *z* axis, then the phase of *a*_Δ_ and the phase of the mw irradiation are defined with respect to the same axis. Accordingly, in this case, the phase of *a*_Δ_ can simply be controlled by the overall phase of the mw pulse sequence. This phase change then corresponds to a *S_z_* rotation, and a phase change of π leads to

$$\begin{array}{c} e^{i\pi S_{z}}\sum_{i}^{N_{I}}\;\frac{B_{i}}{4}\left(a_{\Delta }S^{+}I^{-}+a_{\Delta }^{*}S^{-}I^{+}\right)e^{-i\pi S_{z}}\\ =-\sum_{i}^{N_{I}}\;\frac{B_{i}}{4}\left(a_{\Delta }S^{+}I^{-}+a_{\Delta }^{*}S^{-}I^{+}\right) \end{array}$$ (4)

The effective fields and the nuclear coupling Hamiltonian are not affected by this phase change. In a strict sense, not the whole effective Hamiltonian is inverted. However, the DNP echo formation still occurs if (i) the mismatch of the effective field is not too large and (ii) the timescale of the electron-nuclear transfer is much shorter than any nuclear-nuclear spin dynamics. Condition (i) should be mostly fulfilled for reasonable pulse sequences and narrow EPR spectra, and condition (ii) may be fulfilled by choosing reasonably short DNP echo times. While, in principle, all nuclei take part in the polarization transfer processes during de- and refocusing of the initial electron polarization, the more strongly coupled ones will be more important, as the states containing spin operators of more strongly coupled nuclei will also contribute more to the density operator of the system during the DNP echo sequence. In solid-state NMR, this phenomenon is termed dipolar truncation (*28*).

We should note that the approach of inverting the coupling Hamiltonian is fundamentally different from inverting the effective field. The latter approach changes the sequence from a ZQ to a DQ one. The dynamics then occur in different subspaces, and no echo would be formed. For example, simply inverting the phase of the spin lock in NOVEL does not lead to the formation of a DNP echo.

## MATERIALS AND METHODS

All experiments were conducted on a homebuilt X-band pulsed-EPR/DNP spectrometer [based on the design of Doll *et al.* (*29*)] with a sample of 5 mM trityl (OX063) in a H_2_O:D_2_O:glycerol-d_5_ solution (1:3:6 by volume) at 80 K. This is a common system for pulsed-DNP studies (*30*), although usually with a slightly lower degree of solvent protonation. This is not decisive for the success of the experiments shown in this work. A commercial MD4 electron-nuclear double-resonance probe (Bruker BioSpin) extended with an external tuning and matching circuit was used, and NMR signals were detected with a Spincore iSpin-NMR console (SpinCore Technologies Inc., Gainesville, FL).

### DNP pulse sequence

**Fig. 1.**
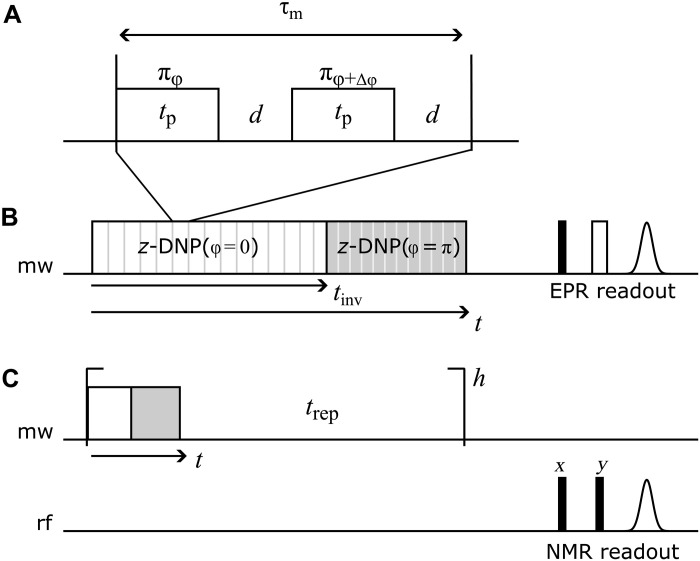
Pulse sequences used in this work. (**A**) Basic DNP building block. Two π pulses of length *t_p_* with a phase difference of Δφ, separated by a delay *d*. The overall length of one element is τ*_m_* = 2(*t_p_* + *d*). The sequence leads to an overall rotation around *S_z_* by an angle of 2Δφ. (**B**) Generation of DNP echo and EPR readout. The basic building block in (A) is repeated for a time *t*_inv_, at which point the overall phase is inverted. The sequence is then continued up to a total time *t*. At this point, the electron polarization is detected via a Hahn echo. The DNP echo forms for *t* = 2*t*_inv_. (**C**) Formation of DNP echo and detection on the nuclear spins. The sequence in (B), excluding the EPR readout, is repeated *h* times, with a repetition time *t*_rep_ on the order of the electron *T*_1,*e*_. The polarization of the bulk protons is then detected with a solid echo. The residual bulk proton polarization is destroyed by a saturation train after each readout. Filled and open pulses in readout corresponds to π/2 and π pulses, respectively. rf, radio frequency.

In principle, any periodic DNP pulse sequence that acts only on the electron spins and generates an effective rotation around *z* can be used. We chose a conceptually simple building block consisting of two π pulses that differ in their phases by Δφ, as illustrated in Fig. 1A. It can be looked at as a special case of TPPM DNP (*24*) with added delays. The delay *d* between the pulses is introduced to make the sequences less susceptible to pulse transients. Overall, the modulation time of the sequence is given by τ_m_ = 2(*t_p_* + *d*). During that time, the nuclear spins rotate around *I_z_* by an angle $\beta_{\text{eff}}^{\left(I\right)}=\omega_{I}\cdot 2\left(t_{p}+d\right)$. For an on-resonance spin packet, the overall electron spin rotation is given by $\beta_{\text{eff}}^{\left(S\right)}=2\Delta \varphi$. The two angles have to match up (modulo 2π). We chose Δφ = (2π−ω*_I_*τ_m_)/2. More concretely, with a nuclear Zeeman frequency of ω*_I_*/2π =−14.787 MHz, we used *t_p_* = 12 ns, *d* = 10 ns, and Δφ = 1.098 ≈ 63°. The exact optimal value for Δφ was set experimentally and could deviate from this prediction by a few degrees. The scaling factor for these values was numerically calculated to be ∣*a*_Δ_∣ = 0.3371. For comparison, NOVEL has a scaling factor of ∣*a*_Δ_∣ = 1.

## RESULTS

DNP echo formation for trityl was observed both on the electron and the nuclear spins. For the electron spins, we used the sequence shown in Fig. 1B. The basic building block described above was repeated for a time *t*_inv_, at which point the overall phase of the building block was changed by π. After a total time *t* and a waiting time of 10 μs, which is long enough for all remaining electron spin coherences to decay but much shorter than the electron *T*_1,*e*_ (about 2 ms), the electron polarization was read out with a simple Hahn echo sequence.

For the nuclear polarization, it is not really possible to detect the protons that are very close to the unpaired electron, unless very low temperatures (≈ 1.5 K) and high electron concentrations are used (*31*). This is due to the low concentration of unpaired electrons, fast nuclear spin relaxation, and hyperfine couplings that exceed the detection bandwidth. However, the electron-nuclear spin dynamics can be imprinted onto the bulk proton magnetization bymaking use of nuclear spin diffusion. For this to occur, one has to repeat the DNP pulse sequence a large number of times; see the pulse sequence in Fig. 1C. The mw pulse sequence in Fig. 1B, excluding the Hahn echo, was repeated *h* = 1000 times with a repetition time of *t*_rep_ = 2 ms. During the waiting times, the nuclear spin polarization diffuses to the bulk protons. This polarization is then read out on the nuclear spins by a solid-echo sequence.

**Fig. 2.**
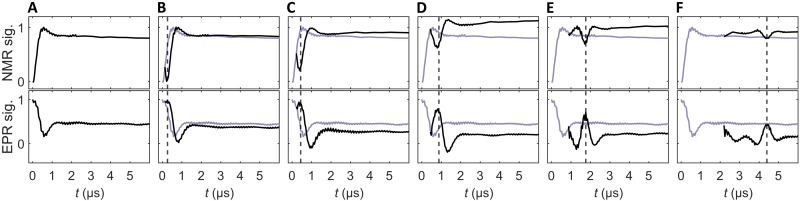
Observation of DNP echoes via NMR and EPR readout. Top: NMR signals. Bottom: Corresponding EPR signals. (**A**) Reference signals for *t*_inv_ = 0. (**B** to **F**) *t*_inv_=132, 220, 440, 880, and 2200 ns. The expected echo position at *t* = 2*t*_inv_ is marked by a dashed line. The reference signal from (A) is shown in gray in the other panels. The apparent stop of small oscillations in the NMR signals after around 2 μs is due to a coarser sampling interval at longer times.

The NMR and EPR signals observed for different phase inversion times *t*_inv_ are shown in Fig. 2. Figure 2A shows the polarization dynamics in the absence of the overall phase change, while Fig. 2 (B to F) was recorded with increasing values for *t*_inv_. One clearly observes the formation of DNP echoes at a time 2*t*_inv_, indicated by dashed lines. For short *t*_inv_ times, i.e., 132 ns, the inversion is essentially perfect, and the nuclear polarization goes back to zero at the expected echo position, while the electron polarization goes back to the initial intensity. At increasingly long *t*_inv_ times, the refocusing is no longer perfect, because the matching condition is not perfectly fulfilled for all spin packets. In addition, higher-order terms of the effective coupling Hamiltonian can also lead to non-perfect refocusing. While the nuclear-nuclear spin couplings might also play a role, we expect this effect to be negligible on the presented timescale. Notwithstanding, the echo formation is still clearly visible in all cases shown here.

One curiosity is that, in some cases, the overall nuclear polarization obtained with the phase inversion is higher than without it, most prominently in Fig. 2D. While this was not the goal of this work, it does give some interesting hints if one wants to obtain as high a nuclear polarization as possible in DNP experiments. These features are qualitatively reproduced in numerical simulations (see the Supplementary Materials). In the presence of mismatches due to electron offsets and mw inhomogeneity over the sample volume, the phase inversion can partially correct some imperfections, as it essentially acts as a composite pulse in the ZQ/DQ subspace.

## DISCUSSION

We have shown that it is possible to generate and observe DNP echoes. This is achieved by choosing a pulsed DNP sequence that forms an effective *z* rotation of the electron spin. Inverting the overall phase of this sequence causes inversion of the electron-nuclear spin coupling term in the effective Hamiltonian responsible for the polarization transfer. This effectively lets the electron-nuclear spin system evolve backward in time. The DNP echo formation can be observed both on the electrons and the nuclei. To supplement the observations in the preceding sections, we here add different aspects that may put the results into perspective and suggest avenues to further investigations and applications.

In principle, any pulse sequence leading to an overall *z* rotation could be used. The recently introduced PulsePol sequence (*20*) should be a good alternative candidate. We did not pursue this in this work because, in our setup, the obtainable electron spin Rabi frequency is only about a factor of 2 to 3 larger than the nuclear Zeeman frequency. This implies that either the pulses would fill a substantial amount of the modulation time or the modulation time would get very long and, thereby, the scaling factor low. In the former case, the PulsePol sequence generates both ZQ and DQ transfer. In typical setups that investigate nitrogen-vacancy centers, the electron spin Rabi frequency is much larger than the nuclear Zeeman frequency of ^13^C, which alleviates this problem.

It might just as well be possible to observe DNP echoes with pulse sequences where the effective field does not point along the laboratory *z* axis. In this case, however, the inversion of the coupling Hamiltonian is more complicated because one has to invert the phase of the pulse sequence in the effective frame, in a time that is short compared to the spin dynamics of the system. If the effective field does not point along *z*, then this cannot be achieved by a simple phase change of the mw pulse sequence. In the case of NOVEL, an infinitely short π pulse along the spin-lock axis would work in theory. In practice, the spin dynamics during the pulse would need to be compensated somehow.

The pulse scheme proposed here inverts the effective hyperfine coupling during DNP, but it leaves the nuclear-nuclear couplings unaffected. In principle, it might be possible to invert the sign of the complete Hamiltonian, although we expect this to be rather challenging to realize in practice.

Serendipitously, we observed that a larger overall nuclear polarization can be achieved with the phase inversion. This observation could be explored further, i.e., by using known composite pulse schemes in the ZQ/DQ subspaces.

We expect that the DNP echo could form the basis for pulse sequences that are used to detect nuclear spins in the vicinity of an electron spin. In the magnetic resonance community, this is known as hyperfine spectroscopy. The DNP echo could be split up, and additional delays and pulses can be added between the two DNP blocks to interfere with the refocusing. Future work will show what additional information can be gained from such experiments. Because the DNP echo and its decay depend on all hyperfine and nuclear-nuclear spin-spin couplings, it might also be possible to infer information about the coupling network around electron spins. Of course, it should also be possible to use DNP echoes in other quantum sensing approaches (*32*), including single-spin readout experiments such as the ones used in nitrogen-vacancy centers (*33*).

## References

[1] A. Abragam, *The Principles of Nuclear Magnetism*, International Series of Monographs on Physics (Oxford Univ. Press, 1961).

[2] R. R. Ernst, G. Bodenhausen, A. Wokaun, *Principles of Nuclear Magnetic Resonance in One and Two Dimensions* (Oxford Univ. Press, 1987).

[3] A. Ponti, A. Schweiger, Echo phenomena in electronparamagnetic resonance spectroscopy. Appl. Magn. Reson. 7, 363–403 (1994).

[4] A. Schweiger, G. Jeschke, *Principles of Pulse Electron Paramagnetic Resonance* (Oxford Univ. Press, 2001).

[5] N. A. Kurnit, I. D. Abella, S. R. Hartmann, Observation of a photon echo. Phys. Rev. Lett. 13, 567–568 (1964).

[6] P. Glorieux, J. Legrand, B. Macke, Microwave echoes in in homogeneous stark fields. Chem. Phys. Lett. 40, 287–291 (1976).

[7] P. R. Berman, J. M. Levy, R. G. Brewer, Coherent optical transient study of molecular collisions: Theory and observations. Phys. Rev. A 11, 1668–1688 (1975).

[8] E. L. Hahn, Spin echoes. Phys. Rev. 80, 580–594 (1950).

[9] H. Schneider, H. Schmiedel, Negative time development of a nuclear spin system. Phys. Lett. A 30, 298–299 (1969).

[10] W.-K. Rhim, A. Pines, J. S. Waugh, Violation of the spin-temperature hypothesis. Phys. Rev. Lett. 25, 218–220 (1970).

[11] W.-K. Rhim, A. Pines, J. S. Waugh, Time-reversal experiments in dipolar-coupled spin systems. Phys. Rev. B 3, 684–696 (1971).

[12] A. Pines, M. G. Gibby, J. S. Waugh, Proton-enhanced nuclear induction spectroscopy. A method for high resolution nmr of dilute spins in solids. J. Chem. Phys. 56, 1776–1777 (1972).

[13] S. R. Hartmann, E. L. Hahn, Nuclear double resonance in the rotating frame. Phys. Rev. 128, 2042–2053 (1962).

[14] M. Ernst, B. H. Meier, M. Tomaselli, A. Pines, Time-reversal of cross-polarization in nuclear magnetic resonance. J. Chem. Phys. 108, 9611–9613 (1998).

[15] A. Abragam, M. Goldman, Principles of dynamic nuclear polarisation. Rep. Prog. Phys. 41, 395–467 (1978).

[16] T. Maly, G. T. Debelouchina, V. S. Bajaj, K.-N. Hu, C.-G. Joo, M. L. Mak-Jurkauskas, J. R. Sirigiri, P. C. A. van der Wel, J. Herzfeld, R. J. Temkin, R. G. Griffin, Dynamic nuclear polarization at high magnetic fields. J. Chem. Phys. 128, 052211 (2008). 18266416 10.1063/1.2833582 PMC2770872

[17] A. S. Lilly Thankamony, J. J. Wittmann, M. Kaushik, B. Corzilius, Dynamic nuclear polarization for sensitivity enhancement in modern solid-state NMR. Prog. Nucl. Magn. Reson. Spectrosc. 102-103, 120–195 (2017). 29157490 10.1016/j.pnmrs.2017.06.002

[18] H. Brunner, R. H. Fritsch, K. H. Hausser, Cross polarization in electron nuclear double resonance by satisfying the hartmann-hahn condition. Zeitschrift für Naturforschung A 42, 1456–1457 (1987).

[19] A. Henstra, P. Dirksen, J. Schmidt, W. Wenckebach, Nuclear spin orientation via electron spin locking (NOVEL). J. Magn. Reson. 77, 389–393 (1969).

[20] I. Schwartz, J. Scheuer, B. Tratzmiller, S. Müller, Q. Chen, I. Dhand, Z.-Y. Wang, C. Müller, B. Naydenov, F. Jelezko, M. B. Plenio, Robust optical polarization of nuclear spin baths using Hamiltonian engineering of nitrogen-vacancy center quantum dynamics. Sci. Adv. 4, eaat8978 (2018). 30182060 10.1126/sciadv.aat8978 PMC6118411

[21] K. O. Tan, C. Yang, R. T. Weber, G. Mathies, R. G. Griffin, Time-optimized pulsed dynamic nuclear polarization. Sci. Adv. 5, eaav6909 (2019). 30746482 10.1126/sciadv.aav6909 PMC6357739

[22] V. S. Redrouthu, G. Mathies, Efficient pulsed dynamic nuclear polarization with the X-inverse-X sequence. J. Am. Chem. Soc. 144, 1513–1516 (2022). 35076217 10.1021/jacs.1c09900

[23] N. Wili, A. B. Nielsen, L. A. Völker, L. Schreder, N. C. Nielsen, G. Jeschke, K. O. Tan, Designing broadband pulsed dynamic nuclear polarization sequences in static solids. Sci. Adv. 8, eabq0536 (2022). 35857520 10.1126/sciadv.abq0536 PMC9286509

[24] V. S. Redrouthu, S. Vinod-Kumar, G. Mathies, Dynamic nuclear polarization by two-pulse phase modulation. J. Chem. Phys. 159, 014201 (2023). 37403849 10.1063/5.0153053

[25] R. Shankar, M. Ernst, P. K. Madhu, T. Vosegaard, N. C. Nielsen, A. B. Nielsen, A general theoretical description of the influence of isotropic chemical shift in dipolar recoupling experiments for solid-state NMR. J. Chem. Phys. 146, 134105 (2017). 28390347 10.1063/1.4979123

[26] A. B. Nielsen, M. R. Hansen, J. E. Andersen, T. Vosegaard, Single-spin vector analysis of strongly coupled nuclei in TOCSY NMR experiments. J. Chem. Phys. 151, 134117 (2019). 31594312 10.1063/1.5123046

[27] A. B. Nielsen, N. C. Nielsen, Accurate analysis and perspectives for systematic design of magnetic resonance experiments using single-spin vector and exact effective Hamiltonian theory. J. Magn. Reson. Open 12-13, 100064 (2022).

[28] M. J. Bayro, M. Huber, R. Ramachandran, T. C. Davenport, B. H. Meier, M. Ernst, R. G. Griffin, Dipolar truncation in magic-angle spinning NMR recoupling experiments. J. Chem. Phys. 130, 114506 (2009). 19317544 10.1063/1.3089370 PMC4435003

[29] A. Doll, G. Jeschke, Wideband frequency-swept excitation in pulsed EPR spectroscopy. J. Magn. Reson. 280, 46–62 (2017). 28579102 10.1016/j.jmr.2017.01.004

[30] G. Mathies, S. Jain, M. Reese, R. G. Griffin, Pulsed dynamic nuclear polarization with trityl radicals. J. Phys. Chem. Lett. 7, 111–116 (2016). 26651876 10.1021/acs.jpclett.5b02720 PMC4935761

[31] Z. Pang, K. Sheberstov, B. A. Rodin, J. Lumsden, U. Banerjee, D. Abergel, G. Bodenhausen, K. O. Tan, Hypershifted spin spectroscopy with dynamic nuclear polarization at 1.4 K. ChemRxiv [Preprint] (2024). 10.26434/chemrxiv-2024-zr8zv. PMC11633758 39661685

[32] C. L. Degen, F. Reinhard, P. Cappellaro, Quantum sensing. Rev. Mod. Phys. 89, 035002 (2017).

[33] A. Gruber, A. Dräbenstedt, C. Tietz, L. Fleury, J. Wrachtrup, C. von Borczyskowski, Scanning confocal optical microscopy and magnetic resonance onsingle defect centers. Science 276, 2012–2014 (1997).

