# Supplementary material for: Observation of dynamic nuclear polarization echoes
Source: Sci Adv. 2024 Oct 18;10(42):eadr2420. doi: 10.1126/sciadv.adr2420 (PMC11488531; doi:10.1126/sciadv.adr2420)
Supplement: Supplementary file 1 — Supplementary Text Fig. S1 [file sciadv.adr2420_sm.pdf]

Science Advances
AAAS

Supplementary Materials for

Observation of dynamic nuclear polarization echoes

Nino Wili *et al.*

Corresponding author: Nino Wili, science@ninowili.ch; Niels Chr. Nielsen, ncn@chem.au.dk

Sci. Adv. **10**, eadr2420 (2024)
DOI: 10.1126/sciadv.adr2420

This PDF file includes:

Supplementary Text
Fig. S1

S1. DERIVATION OF THE EFFECTIVE HAMILTONIAN

A detailed derivation of the effective Hamiltonian during a periodic DNP sequence is already given in reference [23] of the main text. We will give a concise derivation with slightly adjusted notation here for completeness.

We focus on a system with one electron spin and one nuclear spin, as the extension to several nuclei is trivial if only the electron spin is irradiated.

In the laboratory frame, the spin Hamiltonian is given by

$$\mathcal{H}^{(LAB)} = \omega_S S_z + \omega_I I_z + \vec{S} \cdot \mathbf{A} \cdot \vec{I} + \mathcal{H}_{\mathrm{mw}}^{(LAB)} \qquad , \tag{S1}$$

where ω_S and ω_I are the Zeeman frequencies of the electron spin and nuclear spin, respectively, $\mathbf{A}$ is the hyperfine coupling tensor, and $\mathcal{H}_{\mathrm{mw}}^{(LAB)}$ is the microwave irradiation Hamiltonian in the laboratory frame. Next, we go into a rotating frame with $\omega_{\mathrm{mw}} S_z$, and employ the high-field approximation for the electron spin.

$$\mathcal{H}'^{(RF)} = \Delta\omega_S S_z + \mathcal{H}_{\mathrm{mw}} + \omega_I I_z + S_z \left(A_x I_x + A_y I_y + A_z I_z\right) \qquad . \tag{S2}$$

Note that $\Delta\omega_S = \omega_S - \omega_{\mathrm{mw}}$. This corresponds to Equation (1) in the main text for one nucleus. It is customary in the EPR literature to rotate the Hamiltonian around I_z such that the $A_y I_y$ vanishes, such that

$$\mathcal{H}^{(RF)} = \Delta\omega_S S_z + \mathcal{H}_{\mathrm{mw}} + \omega_I I_z + S_z \left(B I_x + A I_z\right) \tag{S3}$$

with $A = A_z$ and $B = \sqrt{A_x^2 + A_y^2}$.

In the next step, we employ an interaction frame transformation with the microwave irradiation and the electron spin offset, i.e.

$$U_S = T \exp(-i \int_0^t (\Delta\omega_S S_z + \mathcal{H}_{\mathrm{mw}}) \mathrm{d}\tau) \tag{S4}$$

$$\begin{aligned} \mathcal{H}'(t) &= U_S^\dagger \left(\omega_I I_z + S_z \left(A I_z + B I_x\right)\right) U_S \\ &= \omega_I I_z + U_S^\dagger S_z U_S \left(A I_z + B I_x\right) \qquad . \end{aligned} \tag{S5}$$

Note that T is the Dyson time-ordering operator. Clearly, only the S_z operator is affected by this transformation. We can rewrite the original operator as

$$S_z \rightarrow \sum_{\chi = x,y,z} R_{\chi z}^{(S)}(t) \tilde{S}_\chi \tag{S6}$$

such that

$$\mathcal{H}'(t) = \omega_I I_z + \sum_\chi R^{(S)}_{\chi z}(t)\tilde{S}_\chi \left(AI_z + BI_x\right) \qquad . \tag{S7}$$

At this point, we remember that we are dealing with periodic irradiation schemes, i.e. $\mathcal{H}_{\mathrm{mw}}(t+\tau_{\mathrm{m}}) = \mathcal{H}_{\mathrm{mw}}(t)$. However, this does *not* imply $R^{(S)}(t)$ is also periodic. One period of irradiation can lead to an overall rotation of the electron spin. This overall rotation can be described by an effective field, implicitly defined by

$$U_S(\tau_{\mathrm{m}}) = T\exp(-i\int_0^{\tau_{\mathrm{m}}}(\Delta\omega_S S_z + \mathcal{H}_{\mathrm{mw}})\mathrm{d}\tau) = \exp\left(\omega^{(S)}_{\mathrm{eff}}\tau_{\mathrm{m}} \quad \vec{z}_{\mathrm{eff}}\cdot\vec{S}\right) \qquad , \tag{S8}$$

where $\omega^{(S)}_{\mathrm{eff}}$ is the (signed) magnitude and $\vec{z}_{\mathrm{eff}}$ is the direction of the effective field, respectively. It is useful to describe the dynamics of a DNP sequence in a frame with the z-axis pointing along the effective field, the operators in this frame are denoted by a tilde

$$\tilde{S}_z = \vec{z}_{\mathrm{eff}}\cdot\vec{S} \qquad . \tag{S9}$$

In order to apply average Hamiltonian theory, the interaction frame Hamiltonian itself should be periodic. This is achieved by going into an interaction frame with the effective field, leading to

$$\mathcal{H}''(t) = -\omega^{(S)}_{\mathrm{eff}}S_z + \omega_I I_z + \sum_\chi R^{(C)}_{\chi z}(t)\tilde{S}_\chi \left(AI_z + BI_x\right) \qquad . \tag{S10}$$

The interaction frame trajectory in this cyclic frame, $R^{(C)}$ can be obtained from the interaction frame trajectory in the rotating frame, $R^{(S)}$ by rotating around the effective field,

$$R^{(C)}(t) = R_z(-\omega^{(S)}_{\mathrm{eff}}t)\cdot R_{\mathrm{flip}}\cdot R^{(S)}(t) \tag{S11}$$

where the rotation matrix R_{flip} aligns the electron spin z-axis with the effective field axis. The rotation matrix $R^{(C)}$ is periodic, and we can write it as a Fourier series

$$R^{(C)}_{\chi z}(t) = \sum_{k=-\infty}^{\infty} a^{(k)}_{\chi z}e^{ik\omega_{\mathrm{m}}t} \qquad . \tag{S12}$$

In a last transformation step, we go into an interaction frame with the resonant part of the nuclear Zeeman interaction, i.e. $k_I\omega_{\mathrm{m}}I_z$, where $\omega_{\mathrm{m}} = 2\pi/\tau_{\mathrm{m}}$ is the modulation frequency of the periodic DNP sequence, and $k_I = \mathrm{round}\left(\omega_I/\omega_{\mathrm{m}}\right)$. This interaction frame leads to

$$\begin{aligned}\mathcal{H}'''(t) = &-\omega^{(S)}_{\mathrm{eff}}S_z + \omega^{(I)}_{\mathrm{eff}}I_z \\ &+\sum_\chi\sum_{k=-\infty}^{\infty} a^{(k)}_{\chi z}e^{ik\omega_{\mathrm{m}}t}(t)\tilde{S}_\chi\left(AI_z + \frac{B}{2}(e^{ik_I\omega_{\mathrm{m}}t}I^+ + e^{-ik_I\omega_{\mathrm{m}}t}I^-)\right) \qquad .\end{aligned} \tag{S13}$$

What remains of the nuclear Zeeman interaction is the nuclear effective field, $\omega_{\text{eff}}^{(I)} = \omega_I - k_I \omega_{\text{m}}$.

This Hamiltonian is now periodic, and the first-order effective Hamiltonian is obtained by only keeping time-independent terms. This is trivial to evaluate at this point, since all time-dependencies are expressed as multiples of the modulation frequency. Only terms with $k = 0$ and $k = \pm k_I$ survive, leading to

$$\begin{aligned}\bar{\mathcal{H}}^{(1)} &= -\omega_{\text{eff}}^{(S)} \tilde{S}_z + \omega_{\text{eff}}^{(I)} I_z \\ &+ A \sum_{\chi} a_{\chi z}^{(0)} \tilde{S}_\chi I_z \\ &+ \frac{B}{2} \left(a_{zz}^{(-k_I)} \tilde{S}_z I^+ + a_{zz}^{(k_I)} \tilde{S}_z I^- \right) \\ &+ \frac{B}{4} \left(a_{-z}^{(-k_I)} \tilde{S}^- I^+ + a_{+z}^{(k_I)} \tilde{S}^+ I^- \right) \\ &+ \frac{B}{4} \left(a_{z}^{(-k_I)} \tilde{S}^- I^- + a_{+z}^{(-k_I)} \tilde{S}^+ I^+ \right) \qquad , \end{aligned} \tag{S14}$$

where we used $a_{\pm z} = a_{xz} \mp i a_{yz}$.

In principle, all the terms in the first-order average Hamiltonian can contribute to the dynamics. However, for many good DNP sequences, the effective fields are quite substantial and/or the terms $A a_{\chi z}$ and $\frac{B}{2} a_{zz}^{(\pm k_I)}$ are quite small. In this case, it is sufficient to keep only the effective fields and the zero- (ZQ) and double- (DQ) quantum terms, i.e.

$$\begin{aligned}\bar{\mathcal{H}}^{(1)} &\approx -\omega_{\text{eff}}^{(S)} \tilde{S}_z + \omega_{\text{eff}}^{(I)} I_z \\ &+ \frac{B}{4} \left(a_{-z}^{(-k_I)} \tilde{S}^- I^+ + a_{+z}^{(k_I)} \tilde{S}^+ I^- \right) \\ &+ \frac{B}{4} \left(a_{-z}^{(k_I)} \tilde{S}^- I^- + a_{+z}^{(-k_I)} \tilde{S}^+ I^+ \right) \qquad . \end{aligned} \tag{S15}$$

Comparing this Hamiltonian to Equation (2) in the main text, we can identify the ZQ and DQ scaling factors

$$a_\Delta = a_{+z}^{(k_I)} \tag{S16}$$

$$a_\Sigma = a_{+z}^{(-k_I)} \tag{S17}$$

S2. ILLUSTRATIVE SIMULATIONS

For some timings, we observed an increased nuclear polarization when inverting the effective coupling Hamiltonian during the polarization transfer. This observation can qualitatively be reproduced in a numerical simulation with a system of one electron and a small,

treatable number of nuclei. We used a simple home-written script in MATLAB, using Hilbert space spin operators, and neglecting relaxation. We used the same sequence parameters as in the main text, *i.e.* $\omega_I/2\pi$ =−14.787 MHz, t_p =12 ns, d =10 ns, and $\Delta\phi = 1.098 \approx 63°$. Additionally, we considered a Gaussian electron offset distribution with $\sigma_{\Delta\omega_S}$ =8 MHz and also a Gaussian microwave amplitude distribution with a relative standard deviation of 5 %. The spin system consisted of one electron and five protons with positions (in Å) $r_1 = (3, 0, 0)$, $r_2 = (-3.2, 0, 0)$, $r_3 = (0, 3.6, 0)$, $r_4 = (0, -4, 0)$, $r_5 = (0, 0, 4.6)$. All coordinates were then rotated around z by $\pi/6$ and around the new y' by $\pi/3$, to ensure a non-vanishing pseudosecular hyperfine coupling. No orientational averaging was performed. The nuclear coordinates are mostly arbitrary, but the distances are roughly in the range of the close proton spins in the trityl OX063.

The resulting nuclear polarization for $t_{\text{inv}} = 0$ and $t_{\text{inv}} = 880$ ns is shown in Fig. S1. Qualitatively, both the (reduced) echo formation, and the slightly increased nuclear polarization in the case of $t_{\text{inv}} > 0$ are reproduced in the numerical simulation.

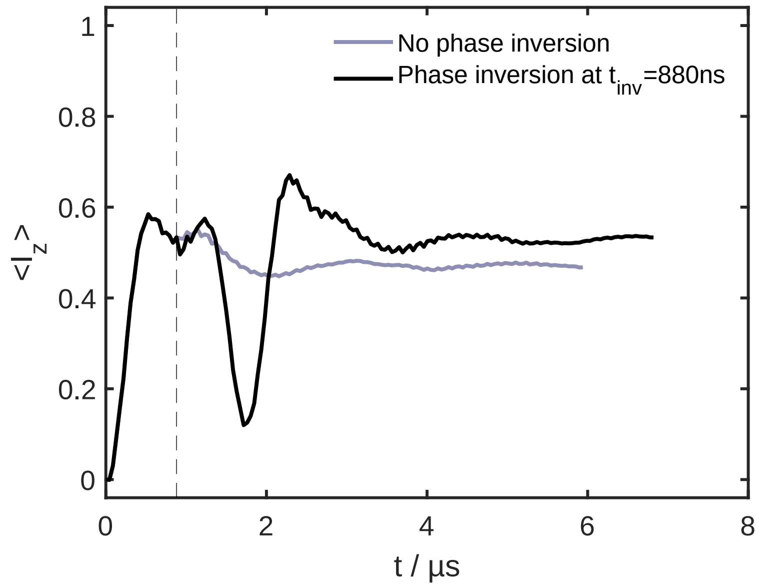

FIG. S1. Simulation of the nuclear polarization $< I_z >$ during a DNP echo. The model system consisted of one electron and five protons. Inhomogeneities in the electron offset and the microwave amplitude were taken into account.
